# Evaluation of a simple method for storage of blood samples that enables isolation of circulating tumor cells 96 h after sample collection

**DOI:** 10.1186/s40709-017-0068-9

**Published:** 2017-09-25

**Authors:** Panagiotis Apostolou, Dimitrios-Athanasios Ntanovasilis, Ioannis Papasotiriou

**Affiliations:** Research Genetic Cancer Centre S.A., Florina, Greece

**Keywords:** Circulating tumor cells, qPCR, Flow cytometry, Transportation, Stability

## Abstract

**Background:**

Minimizing the effects of transportation on the properties of biological material is a major challenge for the scientific community. The viability of cells is important in cases where their study is urgent for evaluation of treatment response or for the study of cancer progression. Circulating tumor cells (CTCs) constitute a cell subpopulation with great importance for oncologists, because of their prognostic value. Detection and isolation of CTCs from blood samples is a routine activity in many laboratories, but concerns exist with regard to the maintenance of the cells during transportation. In this study, experiments were conducted to determine the stability of gene and protein expression in CTCs over a period of 96 h.

**Results:**

Blood samples collected from healthy individuals and patients with cancer were each divided into five aliquots, which were stored at 2–8 °C and analyzed after 0, 24, 48, 72 and 96 h of storage. CTCs from patients and CD45-negative cells from healthy individuals were isolated each day using enrichment protocols, and qPCR was performed to determine expression levels of genes encoding specific biological markers. In addition, cells from breast and colon cancer cell lines were spiked into blood samples from healthy individuals, and these samples were stored and analyzed over a period of 96 h by PCR and by flow cytometry. The markers that were studied included housekeeping genes and genes associated with the response to chemotherapy, as well as genes encoding transcription factors. The results demonstrated that the expression profiles of specific genes and proteins in CTCs were not significantly affected by 72 h of storage. After 96 h of storage, expression of some genes was altered.

**Conclusion:**

The transportation of blood at low temperature (2–8 °C) in the presence of the anticoagulant EDTA can protect CTCs from alteration of gene and protein expression for at least 72 h. Furthermore, under these conditions, CTCs can be detected and isolated 96 h after blood collection.

## Background

In oncology, the detection and isolation of circulating tumor cells (CTCs) are useful procedures for cancer prognosis and prediction of treatment response. These cells constitute a subpopulation of cancer cells that have detached from the primary tumor and circulated through the blood stream, and which can initiate metastatic spread to other organs. A number of methods have been established for detection and isolation of CTCs, and each method has advantages and disadvantages [1]. The CTCs are generally isolated from whole blood using enrichment protocols or flow cytometry [2–5]. A major problem is the stability of this cell population during transportation; collected blood should be handled with care, as any mechanical trauma can be detrimental to the viability of the cells [6]. Specific storage-tube formulations are available, containing different anti-coagulants, but no clear data exist in relation to the stability of samples > 72 h after blood collection.

Although the ability to isolate CTCs from the samples is crucial, it is equally important to demonstrate RNA stability and maintenance of specific antigens. The aim of the present study was to identify CTCs and other cells in blood samples stored at 2–8 °C for 0, 24, 48, 72 and 96 h after collection. In addition to the detection of these cells, expression of relevant RNA and protein markers was evaluated.

## Results

In the first set of experiments, qPCR was used to measure the expression levels of housekeeping genes 18S rRNA, 28S rRNA, *ACTB* and *GAPDH*, as well as the genes *NANOG*, *POU5F1* (*OCT3/4*), *SOX2*, *CD34*, *NES*, *ERCC1*, *DHFR*, *cFOS*, *cJUN*, *cMET* (*HGFR*) and *EGFR*. In the second set of experiments, endpoint PCR was used to identify expression of the housekeeping genes 18S rRNA and *ACTB* and the genes *EPCAM*, *KRT19*, *PECAM1* (also known as *CD31*) and *CDH2* (*N*-*cadherin*).

**Fig. 1:**
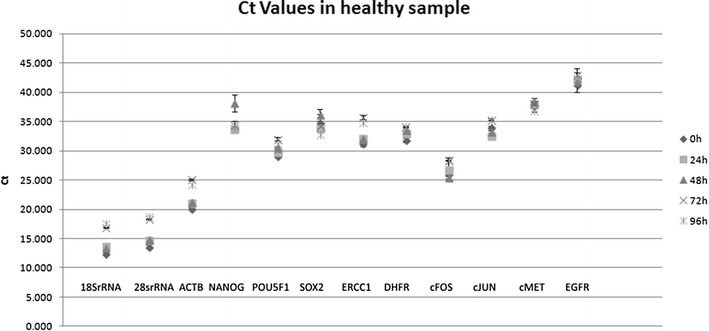
Analysis of gene-expression levels in CD45-negative cells isolated from blood samples taken from a healthy individual and stored for 0–96 h. Gene expression was determined by qPCR, and threshold cycle (Ct) values for entry of reactions into the exponential-amplification phase are shown

**Fig. 2:**
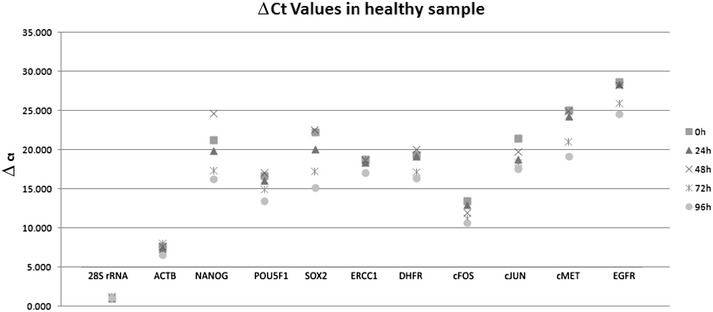
Analysis of gene-expression levels in CD45-negative cells isolated from blood samples taken from a healthy individual and stored for 0–96 h. Gene expression was determined by qPCR, and threshold cycle (Ct) values for specific genes were compared with Ct values for 18S rRNA as an endogenous reference gene, giving ΔCt values

**Fig. 3:**
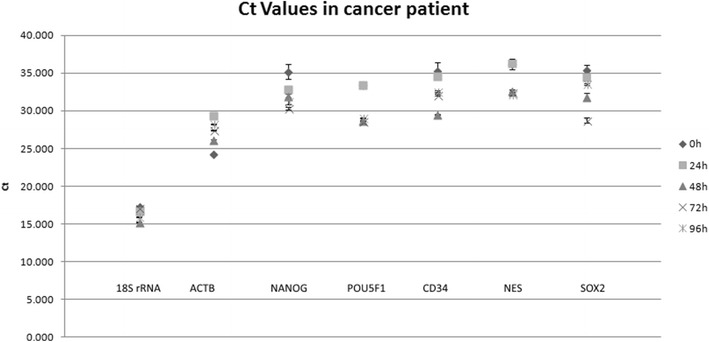
Analysis of gene-expression levels in CD45-negative cells isolated from blood samples taken from a patient with cancer and stored for 0–96 h. Gene expression was determined by qPCR, and threshold cycle (Ct) values for entry of reactions into the exponential-amplification phase are shown

**Fig. 4:**
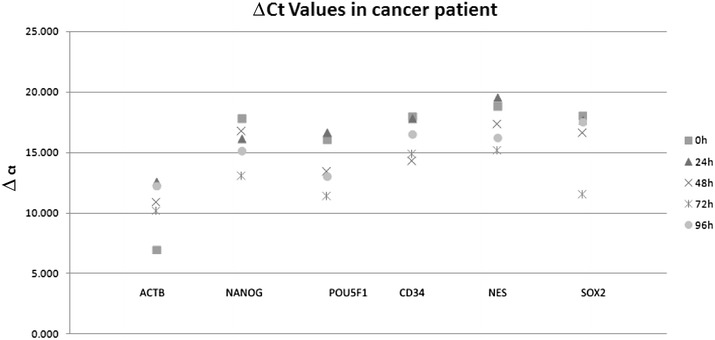
Analysis of gene-expression levels in CD45-negative cells isolated from blood samples taken from a patient with cancer and stored for 0–96 h. Gene expression was determined by qPCR, and threshold cycle (Ct) values for specific genes were compared with Ct values for 18S rRNA as an endogenous reference gene, giving ΔCt values

Expression levels were measured in CD45-negative cells isolated from blood samples from two healthy individuals (Figs. 1, 2). In the samples from one individual, gene expression did not alter significantly from 0 to 96 h, but in the samples from the second individual, qPCR Ct values were significantly different after 96 h, but not after 72 h (*p* = 0.72), compared with the values at 0 h. In the samples from healthy individuals, no expression of *NES* was observed. Gene-expression levels in CTCs isolated from blood samples from two patients with cancer did not alter significantly during the 96 h period of time (*p* = 0.63 and *p* = 0.76) (Figs. 3, 4).

In endpoint PCR experiments, 18S rRNA and *ACTB* were expressed in all samples at all time periods. CD45-negative cells from healthy individuals expressed *PECAM1*, but not *EPCAM*, *KRT19* or *CDH2* (Table 1). The *EPCAM*-positive breast-cancer cells and colon-cancer cells expressed *EPCAM* on all 96 h, whereas they only expressed *KRT19* and *CDH2* from 0 to 72 h (Table 1).

**Table 1:** Endpoint PCR data

Storage time (h)	Gene expression in CD45-negative cells from healthy individuals					
	18S rRNA	*ACTB*	*EPCAM*	*KRT19*	*PECAM1*	*CDH2*
0	+	+	−	−	+	−
24	+	+	−	−	+	−
48	+	+	−	−	+	−
72	+	+	−	−	+	−
96	+	+	−	−	+	−

Storage time (h)	Gene expression in breast-cancer cells spiked into blood from healthy individuals					
	18S rRNA	*ACTB*	*EPCAM*	*KRT19*	*PECAM1*	*CDH2*
0	+	+	+	+	−	+
24	+	+	+	+	−	+
48	+	+	+	+	−	+
72	+	+	+	+	−	+
96	+	+	+	−	−	−

Storage time (h)	Gene expression in colon-cancer cells spiked into blood from healthy individuals					
	18S rRNA	*ACTB*	*EPCAM*	*KRT19*	*PECAM1*	*CDH2*
0	+	+	+	+	−	+
24	+	+	+	+	−	+
48	+	+	+	+	−	+
72	+	+	+	+	−	+
96	+	+	+	−	−	−

Expression of genes was determined electrophoretically by the presence of a product band at the end of PCR. “+” indicates expression, whereas “−” indicates the absence of expression

A blood sample from a healthy individual was tested for expression of CD45 and EPCAM during the 96 h time period (Table 2). The results indicated no significant difference between samples stored for different time periods (*p* = 0.50).

**Table 2:** Flow cytometry data from healthy individuals

	CD45-positive	EPCAM-positive
0 h	85,568.33	0.00
24 h	83,091.33	10.00
48 h	31,815.00	2.33
72 h	38,243.67	0.00
96 h	34,610.00	0.00

Median values for expression of CD45 and EPCAM in blood samples from a healthy individual, determined by flow cytometry

Blood samples from healthy individuals spiked with breast-cancer cells or colon-cancer cells were tested for expression of EPCAM and cMET. Moreover, breast-cancer samples were tested for an additional marker, CD227. EPCAM was expressed in both sample types during the 96 h period, and CD227 was expressed in breast-cancer samples, but cMET was not expressed in any of the samples (Table 3). No significant differences in expression were observed (*p* = 0.79 for breast and *p* = 0.5 for colon cancer cells, respectively).

**Table 3:** Flow cytometry data in cancer samples

	Colon-cancer cells		Breast-cancer cells		
	EPCAM	cMET	CD227	EPCAM	cMET
0 h	111	0	13.67	546.33	0
24 h	542	0	12	648	0
48 h	219.67	0	24	628.33	0
72 h	305.33	0	105	675	0
96 h	593	0	93	446.33	0

Flow-cytometric analysis of expression of biomarkers in colon and breast cancer cell lines up to 96 h after spiking into the blood of a healthy individual

**Fig. 5:**
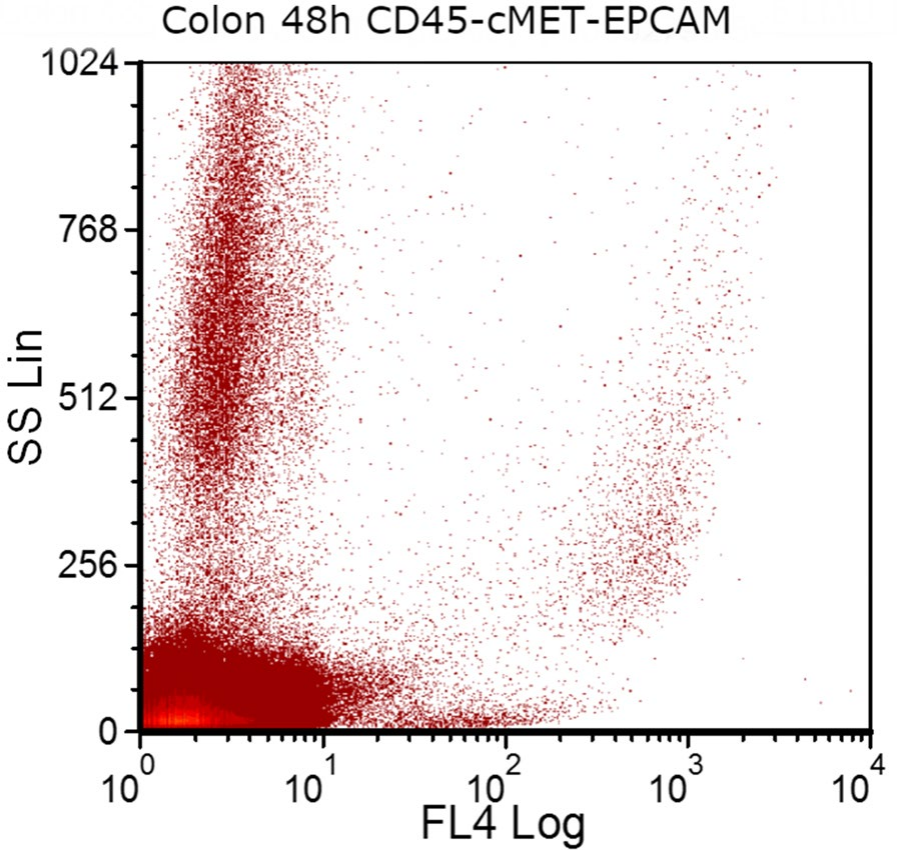
EPCAM (FL4) expression in a colon-cancer cell-line-spiked sample on day 3 of storage. Colon cancer cells from HCT-116 cancer cell line were spiked into whole blood from a healthy donor. After sample staining with CD45, PECAM1 and EPCAM antibodies, EPCAM positive cells were selected by excluding CD45 and PECAM1 positive cells during gating on FCSExpress V3

**Fig. 6:**
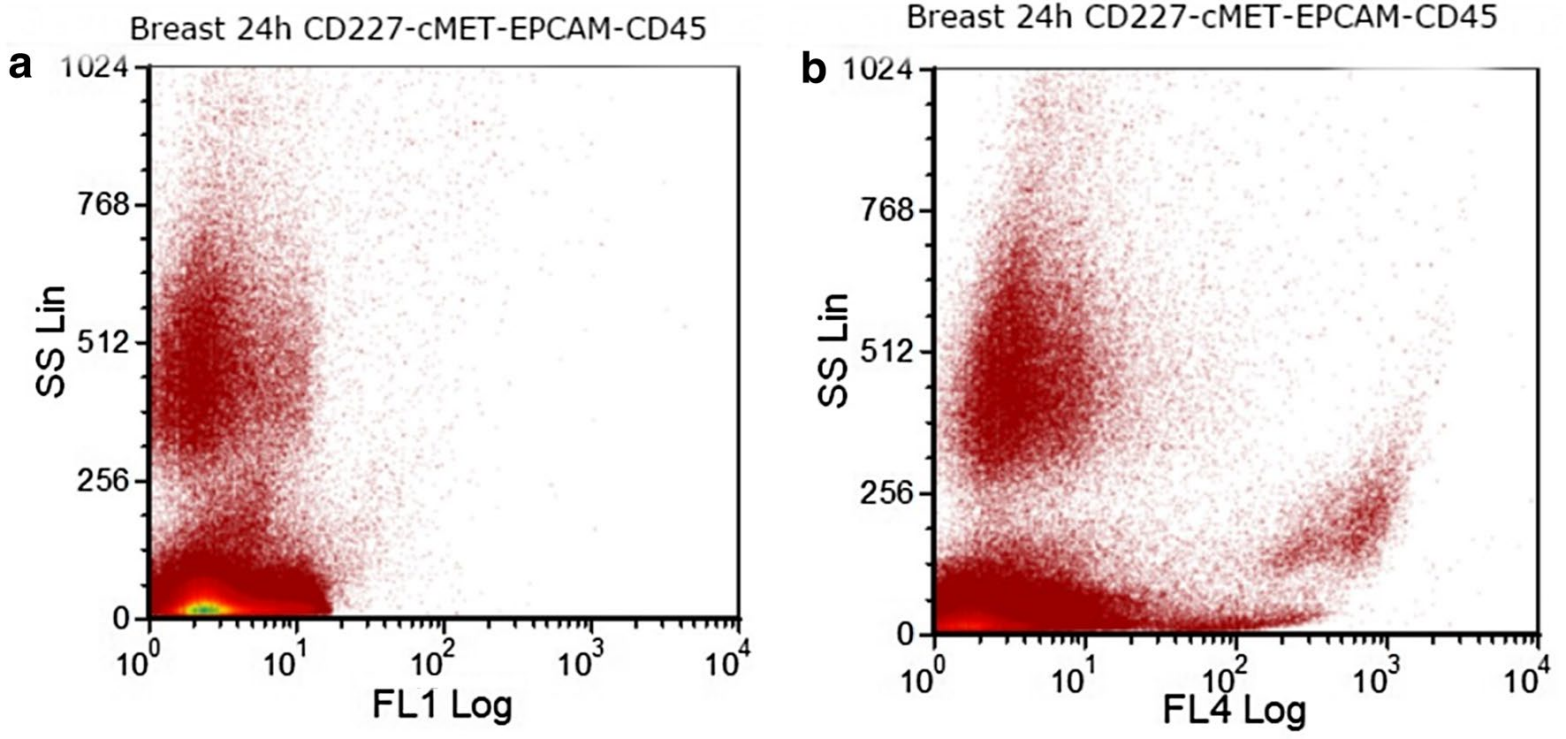
Dot plots showing **a** expression of CD227 (FL1) and **b** expression of EPCAM (FL4) in a breast-cancer cell-line-spiked sample on the day 2 of storage. Breast cancer cells from MDA-MB-231 cancer cell line were spiked into whole blood from a healthy donor. After sample staining with CD45, PECAM1, EPCAM and CD227 antibodies, EPCAM and CD227 positive cells were selected by excluding CD45 and PECAM1 positive cells during gating on FCSExpress V3

**Fig. 7:**
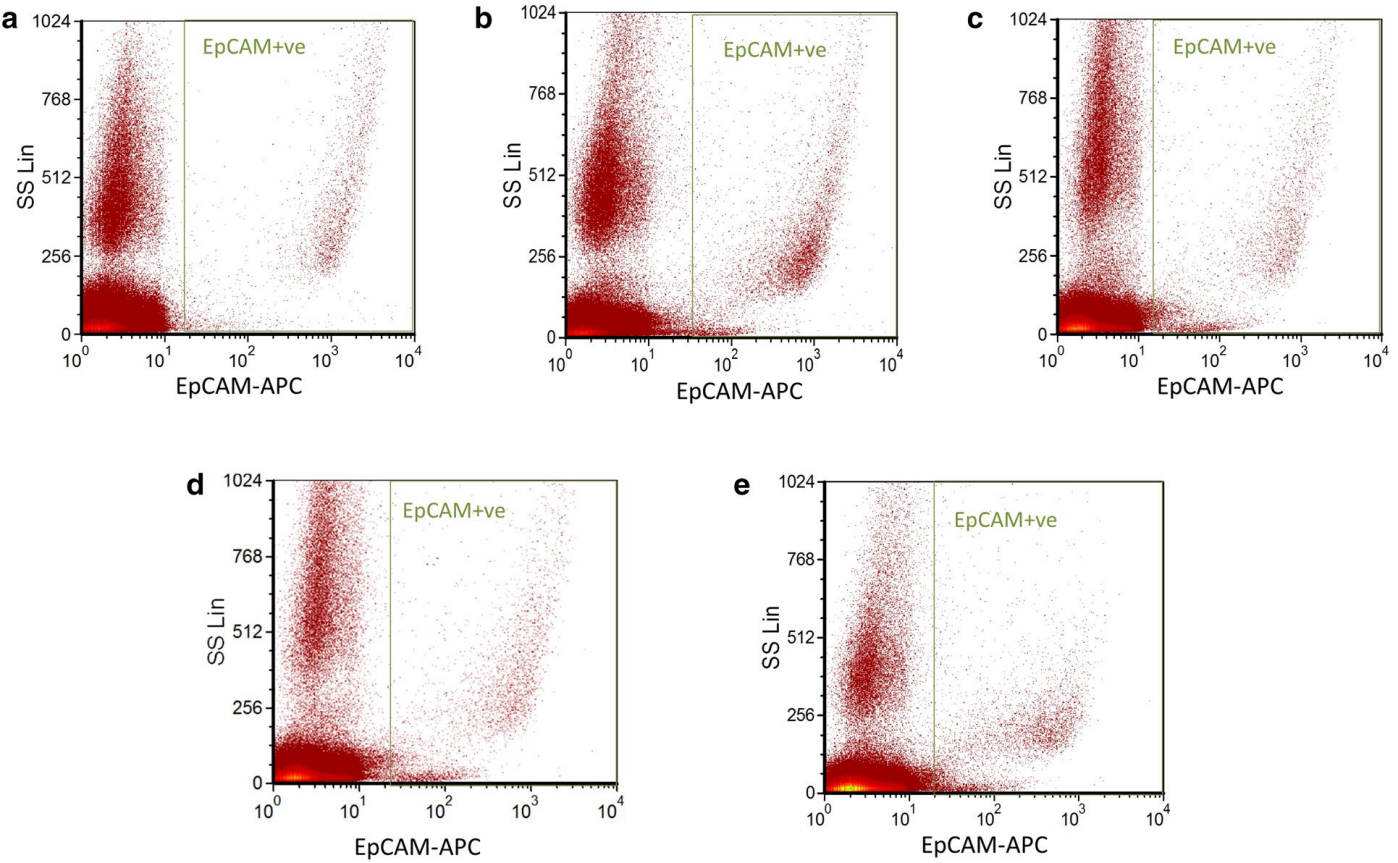
Dot plots showing expression of EPCAM in samples spiked with colon cancer cells from the HCT-116 cell line at **a** 0 h, **b** 24 h, **c** 48 h, **d** 72 h and **e** 96 h. Green gates show the EPCAM^+^ population. Gating on these samples involved excluding CD45 and PECAM1 positive populations

Physical properties of the cell samples, determined by flow cytometry, were compared across the 96 h time period (Figs. 5, 6). Dot plots of side scatter versus EPCAM expression in colon-cancer cells showed that side-scatter properties of the cells changed with storage time, whereas EPCAM expression remained stable (Fig. 7).

**Fig. 8:**
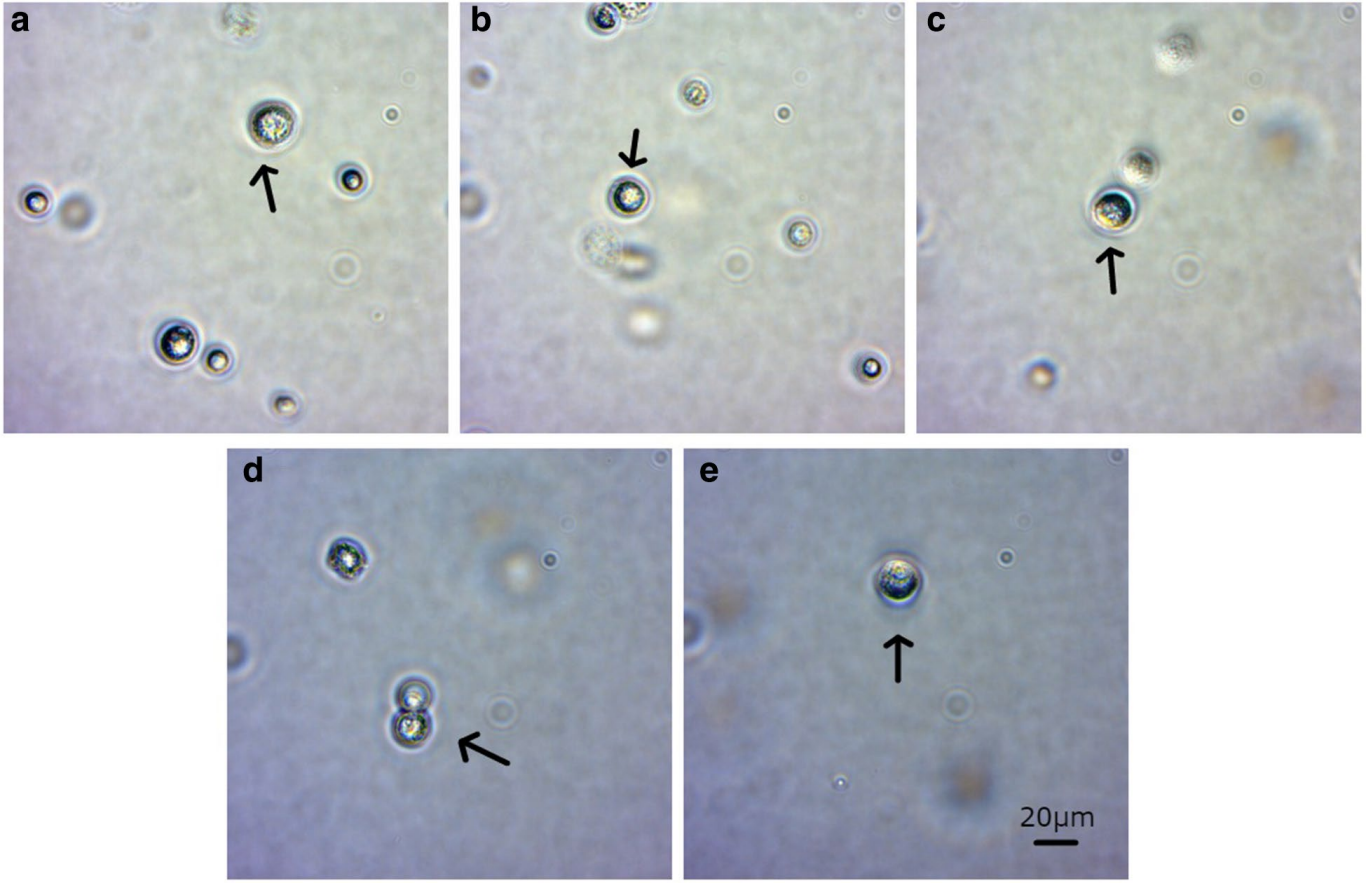
After magnetic bead isolation, cells were viewed under a microscope to determine their phenotype. Pictures were taken at **a** 0 h, **b** 24 h, **c** 48 h, **d** 72 h, and **e** 96 h. Arrows show the presence of CTCs

**Fig. 9:**
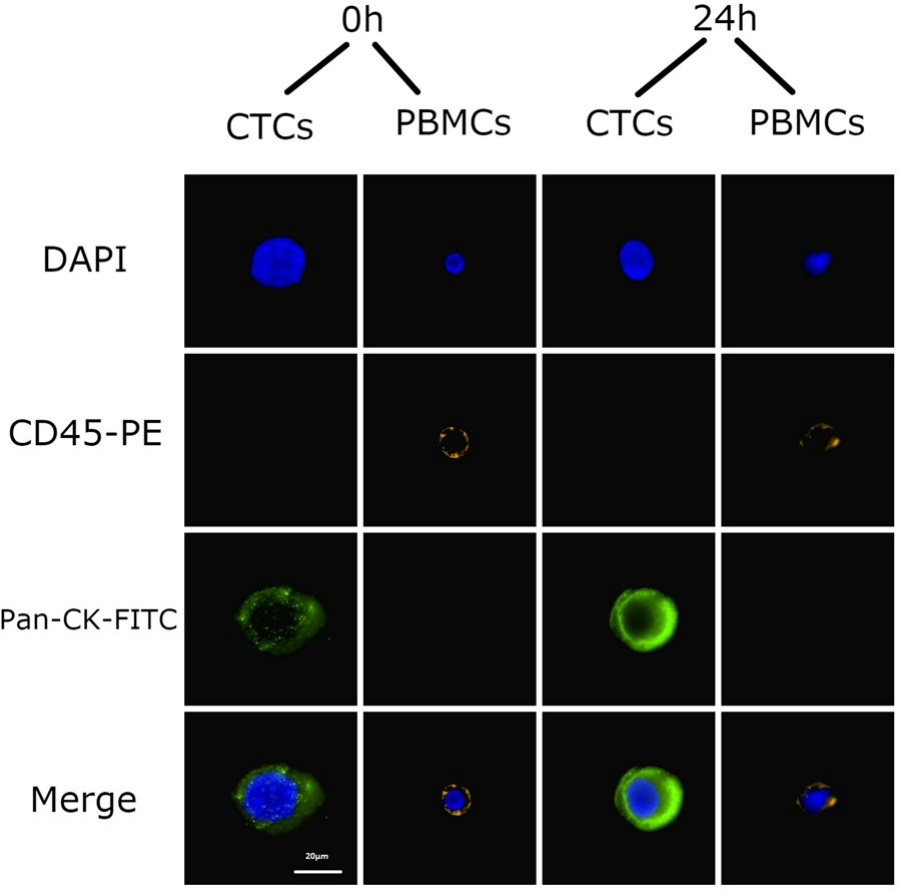
Immunocytochemical analysis of CTCs that were isolated from the samples using Pancytokeratin magnetic beads after 0 and 24 h. DAPI was used to stain the cells’ nucleus. Anti-CD45-PE antibody (orange) was used to stain PBMCs and anti pancytokeratin antibody to stain CTCs

**Fig. 10:**
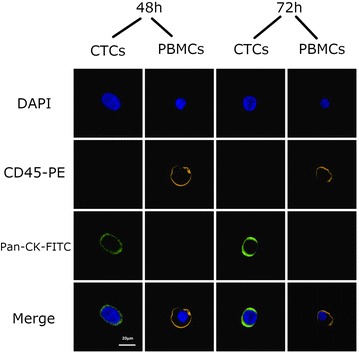
Immunocytochemical analysis of CTCs that were isolated from the samples using Pancytokeratin magnetic beads after 48 and 72 h. DAPI was used to stain the cells’ nucleus. Anti-CD45-PE antibody (orange) was used to stain PBMCs and anti pancytokeratin antibody to stain CTCs

**Fig. 11:**
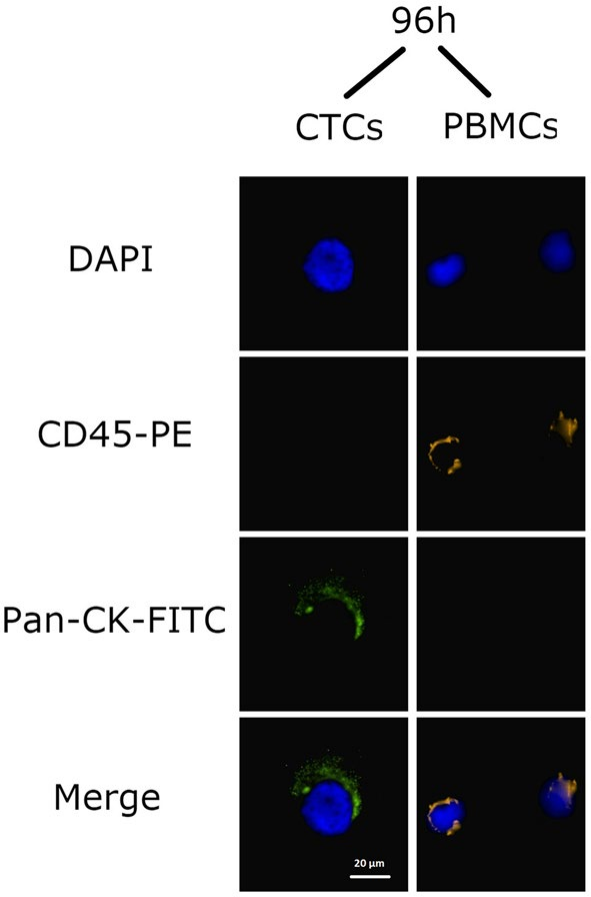
Immunocytochemical analysis of CTCs that were isolated from the samples using pancytokeratin magnetic beads after 96 h. DAPI was used to stain the cells’ nucleus. Anti-CD45-PE antibody (orange) was used to stain PBMCs and anti pancytokeratin antibody to stain CTCs

Regarding the microscopically analysis, the phenotype of the cells did not change after 96 h (Fig. 8). As far as the immunocytochemistry is concerned, no change was also appeared in expression of specific markers (Figs. 9, 10, 11).

## Discussion

Many tests have been validated for routine use in oncology. The transportation of biological material to appropriate laboratories can be problematic and is a source of different opinions between scientists, many of whom claim that the stability of samples alters dramatically over long time periods [6]. Blood is the most widely used biological material, and different transport conditions have been proposed for its maintenance [6]. Appropriate transport conditions can differ depending on the downstream applications. The detection and isolation of CTCs is very important, as they can be used to predict cancer progression and/or resistance to therapy [7]. Therefore, their stability should be evaluated in a time period that represents the transportation time from sampling to analysis. Transportation time and temperature are important variables for determination of optimal transport conditions. Many researchers claim that samples should not be stored for longer than 6 h at temperatures between 20 and 25 °C [8]. However, according to Hankinson et al., temperature has no significant effect on biochemical markers [9]. Other groups have demonstrated that the preservation of CTCs in blood samples can be promoted by using a sugar-based cell-transportation solution, in which viability can be maintained for > 72 h [10]. Proprietary storage tubes called Cell-Free DNA™ BCT devices are available; in these tubes, CTCs are stable for at least 4 days at room temperature [11]. CellSave Preservative Tubes are also approved for stabilization of CTCs for up to 96 h at room temperature. EDTA is the most widely used anticoagulant agent, and is suitable for preservation of cells in blood samples, as well as cell-free DNA [12].

The aim of the present study was to demonstrate that CTCs can be detected and isolated after storage for up to 96 h. According to our protocol, blood samples should be stored in tubes containing EDTA, at a temperature between 2 and 8 °C. These conditions should preserve gene expression for up to 72 h, but after 96 h of transportation, changes in expression of specific markers may be observed.

Our experimental data indicated that stored CTCs or CD45-negative cells do not lose expression of specific markers that may be correlated with stemness (such as *NANOG* and *POU5F1*) [13], or resistance to chemotherapeutic agents (such as *ERCC1* and *DHFR*) [14, 15]. The change of ΔCt parameter was also not affected and the results of gene expression were accurate. Furthermore, the study of genes implicated in key pathways, like the mitogen-activated protein kinase (MAPK) pathway [16], indicated that genes located in the nucleus were not affected. Our results indicated that RNA can be isolated from CTCs for prognostic or diagnostic purposes even 96 h after blood collection.

Our results also suggested that distinctive patterns of gene expression in cancer cell lines spiked into blood samples from healthy individuals were maintained up to 72 h with this storage protocol.

Our flow-cytometric data indicated that protein expression of markers for identification of CTCs, as well as CD45 (an antigen expressed by all leukocytes) [17], did not change significantly over the 96 h time period. Dot plots of forward scatter versus side scatter demonstrated no apparent changes to the physical properties of the cells [18]. The maintenance of biomarker expression indicated that samples stored under these conditions are sufficiently stable for shipping and further analysis, without increasing the possibility of false-positive results.

Taking everything into consideration, it is clear that the transportation of genetic material is very important when CTCs analysis is required. There are different transportation solutions, which should not be ignored depending on the study we need to perform. For example, if cfDNA analysis is needed, specific tubes protecting this type of material, are essential. However, in this situation, there is no ability to protect the mRNA and therefore the proteins for further studies [11]. The solution of using sugar based transportation solution is quite promising, since CTCs are viable after 6–7 days. If the enumeration of CTCs is required, then the last transportation solution is suggested, since no specific transportation conditions are required [10]. However, even in this case, there are no data regarding the stability of gene and protein expression. The use of collection tubes filled with EDTA and the transportation under specific conditions of temperature seems the most promising, since apart from the viability of the CTCs it enables further study at both gene and protein level. In addition, the transportation does not require specialized laboratory consumables as well as specific tubes.

## Conclusion

The present study aimed to demonstrate that CTCs can be detected and isolated after storage for at least 72 h. Blood was stored in tubes containing EDTA at temperatures between 2 and 8 °C. With storage for 72 h, gene expression in isolated cells was stable, but at 96 h, changes in expression of specific markers were observed. On the basis of these results, future studies could expand this analysis to include more samples (including other types of cancer) and additional biomarkers.

## Methods

### Sample collection

Blood samples (40 ml) from two patients with cancer and two healthy donors were collected in sterile 50 ml Falcon tubes (4440100, Orange Scientific, Braine-l’Alleud, Belgium) containing 7 ml of 0.02 M EDTA (E0511.0250, Duchefa Biochemie B.V., Haarlem, The Netherlands) as an anticoagulant. One patient was a 45-year-old woman with stage II breast cancer, and the other patient was a 54-year-old man with stage III colorectal cancer. The healthy individuals were a 30-year-old man and a 28-year-old woman. The samples were placed on a roller for 30 min, divided to five clean 50 ml Falcon tubes and stored at 2–8 °C. The study was performed from April to September 2016.

### Cell lines

A human breast-cancer cell line (MDA-MB 231) and colon-cancer cell line (HCT-116) were obtained from the European Collection of Cell Cultures (ECACC-HPA cultures, Salisbury, UK). Cells were cultured in 75 cm^2^ flasks (5520200, Orange Scientific) at 37 °C in a 5% CO_2_ atmosphere, in the recommended media supplemented with the appropriate amount of heat-inactivated fetal bovine serum (10106-169, Invitrogen, NY, USA) and 2 mM l-glutamine (G5792, Sigma-Aldrich, Munich, Germany). Approximately, 3,750,000 cells were isolated and divided into five 15 ml Falcon tubes each containing 1.5 ml of blood from healthy individuals, with EDTA anticoagulant (with a total of ~ 750,000 cancer cells in each tube).

### Sample preparation

Whole-blood samples were centrifuged for 20 min at 2500×*g* with 4 ml polysucrose solution (Biocoll separating solution 1077, Biochrom, Berlin, Germany). Mononuclear cells, lymphocytes, platelets and granulocytes were collected after centrifugation and washed with phosphate-buffered saline (PBS) (P3813, Sigma-Aldrich). The cells were incubated in lysis buffer [154 mM NH_4_Cl (31107, Sigma-Aldrich), 10 mM KHCO_3_ (4854, Merck, Darmstadt, Germany), and 0.1 mM EDTA in deionized water] for 10 min to lyse the erythrocytes. Samples were then centrifuged as above and washed with PBS. Cells from the healthy donors (non-cancer) were incubated at 4 °C for 30 min with CD45 magnetic beads (39-CD45-250, Gentaur, Kampenhout, Belgium), whereas those from patients with cancer were incubated with pan-cytokeratin beads (recognizing CK4, CK5, CK6, CK8, CK10, CK13 and CK18) (5c-81714, Gentaur) at 4 °C for 30 min. Following incubation, the samples were placed in a magnetic field to collect microbead-bound cells for pan-cytokeratin and negative selection was performed for CD45 cells, which were washed with PBS. Molecular analysis was performed on the isolated CD45-negative cells (non-cancerous) and the pan-cytokeratin-positive cells (cancerous).

### Molecular analysis

Total RNA from cultured cells was extracted using an RNeasy Mini Kit (74105, Qiagen, Hilden, Germany). Total RNA samples were evaluated spectrophotometrically, and 1 µg of each RNA sample was used as a template for cDNA synthesis using a PrimeScript RT Reagent Kit (RR037A, Takara, Beijing, China). Real-time qPCR was then performed using KAPA SYBR Fast Master Mix (2 ×) Universal (KK4618, KAPA Biosystems, MA, USA) in a final volume of 20 μl. Specific primers for each marker and for reference genes were designed using Gene Expression 1.1 software (Genamics, New Zealand). Length parameter was set between 20 and 25 bp, %GC 40–60, Tm Range 57–60 °C, 3′ End Stability from − 3 to − 12, 5′ End Stability from − 6 to − 9 and finally without ΔG Dimer and ΔG hairpin. Primer sequences were evaluated by BLAST searching to exclude those that would amplify undesired genes (Table 4). The PCR program was as follows: initial denaturation at 95 °C for 2 min followed by 45 cycles of denaturation at 95 °C for 10 s and annealing at 59 °C for 30 s. Melting-curve analysis was performed from 65 to 95 °C with 0.5 °C increments for 5 s at each step. The qPCR products were run on agarose gels and visualized, to validate the results. ΔCt value was used for analysis of experiments. The ΔCt calculates the relative expression of a gene of interest in relation to another gene (adequate reference gene).

**Table 4:** Primers used in PCR experiments

Gene	Forward primer (5′–3′)	Reverse primer (5′–3′)
18S rRNA	TGCCCTATCAACTTTCGATGGTAGTC	TTGGATGTGGTAGCCGTTTCTCA
28S rRNA	CGGGTAAACGGCGGGAGTAAC	TGGCTGTGGTTTCGCTGGATAG
*ACTB*	GCCCTGGACTTCGAGCAAGAGA	CAGGAAGGAAGGCTGGAAGAGTG
*NANOG*	CGTGTGAAGATGAGTGAAACTG	GGATGGGCATCATGGAAA
*POU5F1*	AGGAAGCTGACAACAATG	ACTCGGTTCTCGATACTG
*CD34*	CCCATGCTGGAGGTGACATCTC	CCAGGGAGCCGAATGTGTAAAG
*NES*	GAGACACCTGTGCCAGCCTTTCTTA	CTGGGCTCTGATCTCTGCATCTACAG
*SOX2*	CTCGCCCACCTACAGCAT	GCTGGCCTCGGACTTGAC
*cFOS*	CCTTCACCCTGCCTCTCCTCAAT	GCCTGGATGATGCTGGGAACA
*cJUN*	CCAACTCATGCTAACGCAGCAGTT	ACCCTTGGCTTTAGTTCTCGGACAC
*cMET*	AACAGGTGCAAAGCTGCCAGTG	GCACGCCAAAGGACCACACAT
*DHFR*	AGTCAGCGAGCAGGTTCTCATTGA	TGGACTATGTTCCGCCCACACA
*EGFR*	CCCCTGACTCCGTCCAGTATTGA	CTTTTCCTCCAGAGCCCGACTC
*ERCC1*	GCTACCACAACCTGCACCCAGACT	GCAGTCGGCCAGGATACACATCT
*EPCAM*	ACTGCTGGGGTCAGAAGAACAGAC	TCTGAAGTGCAGTCCGCAAACTT
*PECAM1*	GGTCAGCAGCATCGTGGTCAAC	GGATGGAGCAGGACAGGTTCAGT
*CDH2*	CGTGAAGGTTTGCCAGTGTGACTC	TTATCCCGGCGTTTCATCCATAC
*KRT19*	AGCGGCTCATGGACATCAAGTC	AGGACAGCAGAAGCCCCAGAG

The above table presents the sequence of the primers used in the present study. The primers are presented in 5′ to 3′ sequence

For the endpoint PCR, cDNA was amplified using GoTaq G2 Flexi DNA Polymerase (M7805, Promega, WI, USA) with the follow PCR program: initial denaturation at 95 °C for 5 min followed by 35 cycles of denaturation at 95 °C for 15 s, annealing at 59 °C for 15 s and extension at 72 °C for 30 s, along with a final extension step at 72 °C for 10 min. The reaction products were separated by electrophoresis on agarose gels and visualized.

The final primer concentration was 400 nM in both qPCR and endpoint PCR. In all sets of reactions, cDNA from Universal Human Reference RNA (740000-41, Agilent, CA, USA) was used as a positive control. Template-free and negative controls were also used in all experiments. All the reactions were performed in triplicate.

### Flow-cytometry sample preparation and staining

Aliquots (0.5 ml) from the previously prepared 15 ml Falcon tubes, containing ~ 250,000 cells from a cancer-cell line spiked into normal whole blood, were analyzed by flow cytometry. Red blood cells were lysed using ammonium chloride, and samples were then stained with the antibodies CD45-PC7 (25-0459-42, eBioscience, Wien, Austria) and CD31-RPE (MCA1738PE, Abd Serotec, Segrate, Italy), which were used to exclude CD45^+^ cells and endothelial cells, respectively, during analysis of the data. The antibody EPCAM-APC (324208, Biolegend, CA, USA) was used to identify cancer cells of epithelial origin and CD227-FITC (MCA1742F, Abd Serotec) was used to further identify breast-cancer cells expressing mucin-1. Flow cytometry was performed on a Beckman Coulter FC500 flow cytometer equipped with a 488 nm blue laser and a 633 nm red, air-cooled laser. Each experiment was performed in triplicate.

### Gating strategy and data analysis

Data analysis was performed using the FCSExpress software (DeNovo Software V3). Gating was performed with dot plots. To exclude non-target cells, a forward scatter/side scatter (FS/SS) dot plot was used to exclude debris, an SS/CD45 dot plot was used to exclude all white-blood-cell populations, and an SS/CD31 plot was used to exclude all CD31^+^ endothelial cells. To select the EPCAM^+^ and CD227^+^ populations, gates were drawn on SS/EPCAM and SS/CD227 dot plots to select the positive populations.

### Microscopy evaluation

The isolated cells were evaluated microscopically during the 96 h. For each sample, cells were plated in microscopy slides, visualized in an inverted Zeiss Microscope (Primovert) and captured by using the Axiocam and the ZEN software (ZEISS, Germany). The exposure and time frame of the camera were set automatically, according to manufacturer’s guide (zoom at 10 ×).

## Immunocytochemistry

Immunocytochemistry assays were performed for isolated cells for 0, 24, 48, 72 and 96 h. The cells were fixed with formaldehyde 4% (1040032500, Merck) for 20 min. Afterwards, they were washed with water and placed on microscope slides (J1800AMN2, Thermo, CA, USA). Cells were incubated in blocking buffer (1 × PBS, 10% FBS, 0.3% Triton-X) for 45 min and washed twice with washing buffer (1 × PBS, 0.1% BSA). Antibodies were added and incubated overnight at 2–8 °C. They contained CD45-PE (177018, Beckman Coulter, Nyon, Switzerland) and Monoclonal Anti pan-Cytoceratin (SAB4700666, Sigma-Aldrich) labeled with FITC (ab102884, Abcam) according to manufacturer’s instructions. Slides were washed twice with wash buffer and finally DAPI II Counterstain (06J50-001, Abbott Molecular, Illinois, USA) was added before visualization of slides. The samples were visualized under a Nikon Eclipse 50i equipped with a mercury lamp and captured with CytoVision software (Leica Biosystems).

### Statistical analysis

The qPCR results were tested for normal distribution with the Kolmogorov–Smirnov test. One-way ANOVA tests were also performed on the qPCR data to test for significant differences between the various samples. A significant *p* value was defined at < 0.05 level. Statistical analysis was performed with PAST version 2.10 [19].

## Abbreviations

CTCs: circulating tumor cells
qPCR: quantitative polymerase chain reaction
MAPK: mitogen-activated protein kinase
SS: side scatter
FS: forward scatter
